# Behaviour of Children and Adolescents and the Use of Mobile Phones in Primary Schools in the Czech Republic

**DOI:** 10.3390/ijerph18168352

**Published:** 2021-08-06

**Authors:** Kamil Kopecký, Francisco-Domingo Fernández-Martín, René Szotkowski, Gerardo Gómez-García, Klára Mikulcová

**Affiliations:** 1 Centre for Prevention of Risky Virtual Communication, Faculty of Education, Palacký University, CZ-779 00 Olomouc, Czech Republic; kamil.kopecky@upol.cz (K.K.); rene.szotkowski@upol.cz (R.S.); klara.mikulcova1@gmail.com (K.M.); 2 Department of Developmental and Educational Psychology, Campus de Cartuja s.n., 18071 Granada, Spain; fdfernan@ugr.es; 3 Department of Didactics, School Organization, University of Granada, 18071 Granada, Spain

**Keywords:** mobile phone, children, behaviour, schools, internet threats

## Abstract

Today’s young people spend most of their time in contact with mobile devices. Their excessive use carries many risks, such as addiction, cyberbullying and social disruption. Based on this, this study analysed the mobile phone use of young Czechs between 7 and 17 years old (*n* = 27.177) and assessed the differences in their behaviour according to the mobile device use policies of their schools. The results show that the use of mobile phones was linked to the one of the social networks, YouTube and videogames for the most part. Similarly, those young people who had them at school preferred to use them, instead of practicing sports or social activities. On the other hand, in the centres in which the use of mobile phones was prohibited, they felt bored and without activities to do. Therefore, it will be necessary for schools to implement educational policies that encourage activities and areas of social interaction in the school, especially during recess. However, at the same time, it is recommended not to prohibit the use of technological devices in the educational centre, since this fact encourages students to use them secretly and increases their desire to use them. To this end, its use in the classroom is advocated from an educational perspective, thus promoting collaborative learning and increasing student motivation.

## 1. Introduction

Since the end of the 20th century, the intensification and scope of the use of new electronic media by children and young people has been growing systematically. However, this mainly relates to the entertainment and communication functions offered by these types of devices [1]. Today, the mobile phone has become an indispensable element in the lives of children and adolescents [2]. Specifically, as the International Data on Youth and Media reports for 2019 indicated, there has been a dizzying increase on the use of mobile phone, especially at younger ages [3,4], which places the start of the interaction with this device from the age of 7 [5]. This is due to the changes in lifestyles being experienced by young people as a result of the advent of the information society [6].

Thus, there has been an enormous increase in children’s and adolescents’ use of mobile phones by in the early stages. This increase is associated with two variants of its use, the communicative skill that focuses on calls, messages and, mostly, use of social networks [7] and, on the other hand, the recreational one, which has to do with the viewing of multimedia material and the practice of several mobile games [8]. Similarly, the arrival of events such as the YouTuber phenomenon, which turns young people into not only receivers but also creators of their own content, has led to an increase in the consumption rates of mobile devices in recent years [9,10].

Children and adolescents seek to discover, explore and investigate the potential of these devices, this being, in most cases, in an autonomous way. This can lead them to experience age-inappropriate situations. Lack of maturity and skills to manage inappropriate content have even led some of them to experience psychological, physical discomfort or specific behaviours, such as phubbing or nomophobia [11,12,13,14,15].

There is a debate about the permissiveness of mobile phone use in schools. There are several experts who advocate the advantages of mobile devices in schools, arguing their novelty, the attraction they produce on students and the wide range of educational offerings with which they provide the teacher, by providing new teaching/learning strategies [16]. Based on these ideas, multiple studies have incorporated these devices into their educational practice, obtaining a successful response from students [17,18,19,20].

On the other hand, among those who advocate the disadvantages of mobile phone use in the educational field, they distinguish several identified risks associated with mobile phone use in the youth sector. Among these, the threat that generates the highest fear is its enormous addictive potential [21,22]. This refers to the notion that digital devices physically and psychologically stimulate the human brain. This is where receiving a “Like” on Facebook or Instagram becomes a priority to be achieved by today’s youth [23,24]. Several experts have analysed the potential use of these devices throughout the day in children and adolescents [25], concluding that it is necessary to know the attitudes of use of young people in each context in order to promote preventive attitudes that would encourage a responsible use of these devices.

Thus, there is also a vision in which the popularity of mobile phones is not synonymous with making good use of them. In relation to this idea, there is research that shows the relationship between the use of mobile phones on school premises and lack of concentration, of reflection and criticism, or poor school performance by students, which seriously affects their school performance as a whole [26,27,28,29]. Likewise, the literature also provides the possible relation between the use of these devices in the school and the increase of cases of school bullying and cyberbullying [30,31,32]. This has led to a ban on the use of mobile phones in schools in several countries. However, there are several experts who claim that banning the use of mobile devices in schools is not the solution to eradicate the events of addiction that are being observed in society [33].

Therefore, there is a clear need to identify which are the attitudes towards mobile phone use in each context, in order to provide relevant information that leads to the development and subsequent implementation of measures to solve and prevent this phenomenon.

As a result of this idea, this research aims at understanding attitudes towards mobile phone use by children and adolescents, specifically in the Czech Republic, in order to provide a general framework of information on the child–mobile phone relationship both in their everyday life and in the school setting. This objective can be stratified into the following specific subobjectives:

- Which activities are the most frequented by young people when interacting with the mobile device.
- Which activities are carried out by young people during school breaks.
- Which activities are most frequented by young people in the centres where the use of mobile devices is forbidden.

## 2. Materials and Methods

### 2.1. Research Identification

The research Czech Children in the Cyberworld was carried out by the Centre for the Prevention of Virtual Risk Communication at the Faculty of Education of Palacký University in Olomouc, in cooperation with O2 Czech Republic. It is based on the research projects on risk behaviour of kids and adults in the on-line sphere, completed by the very same team in 2015–2018 and, in particular, on the following studies: The risks of Internet communication IV (2014) and Sexting and risk behaviour of Czech Kids in Cyberspace (2017), complementing them with new findings, something unique in the Czech Republic. The analysis of the dataset and the interpretation of the results were carried out in collaboration with the AREA research group (HUM-672), Department of Didactics and School Organization of the University of Granada (Spain).

The study was funded by O2 Czech Republic within the framework of the so-called contract research. No public or EU funding was obtained.

The study was conducted according to the guidelines of the Declaration of Helsinki and ap-proved by the Ethics Committee of Palacky University of Olomouc (REF:8012/2020), 2 December 2020.

### 2.2. Procedure

We selected an anonymous online survey as the core research tool. It was distributed to primary/ first years of secondary schools in all regions of the Czech Republic, where data were collected. The data were collected from 1 February 2019 to 1 May 2019. The evaluation and interpretation of the part results was completed in the following weeks. Prior authorisation was sought from each of the students so that they could participate in the research. The Cronbatch alpha coefficient was valid (α = 0.879). The survey was distributed through the Google Forms software.

The questions asked in the survey aimed at exploring which habits they had in their daily school life and in their free time, as well as during class periods. The questions mainly focused on how they used their cell phones and how often they did so (chatting on their cell phones, using social networks, etc.). As for data analysis, the relevant statistics were processed and performed using the Statistica software.

### 2.3. Participants

A total of 27,177 respondents aged 7–17 from all Czech regions participated in the research and boys constituted 49.83% of the sample. The average age of all respondents was 13.04 years (median 13, modus 12, variance 4.34). The sample configuration was carried out through simple random sampling, in which different schools were randomly selected from the different Czech regions to participate in the completion of the questionnaire. Subsequently, those pupils whose parents gave their consent to participate formed the research sample. Responses came mainly from schools in Moravskoslezský, Olomoucký and Středočeský (Czech Republic), with a response rate of 75.3% of their total schools.

## 3. Results

### 3.1. A. Children and Mobile Phones

In our research, we focused on active usage of mobile phones by children. We wanted to know whether a child had a mobile phone with Internet access without the need of Wi-Fi connection (e.g., through 3G, 4G, LTE, etc.). Over half of the children (59.1%) confirmed that they had permanent Internet access on their mobile phone and, therefore, did not have to rely on Wi-Fi (Table 1).

The most frequent activity reported by children was making/receiving phone calls (72%), followed by typing and sending messages on on-line services (Facebook Messenger, WhatsApp, etc.) (66%). The following place was taken by watching videos on YouTube and typing SMS messages.

#### 3.1.1. Mobile Phones in Schools

A question that echoes strongly in the Czech Republic, as well as in other European countries, is how to regulate the children’s use of mobile phones in schools—whether to ban mobile phones during lessons and breaks or to limit the ban only to lessons and not breaks (see the opinion of the Czech School Inspectorate). Therefore, we asked children about their experience with mobile phone restrictions and how this issue was dealt with in the school they attended (Table 2).

The majority of children (53.3%, 14,486 children) were permitted to use mobile phones during recess at school and this was not permitted during classes. However, at the teacher’s instruction, they were also allowed to use their mobile phones during lessons—the mobile phone becomes a learning aid/tool. However, a significant number of children (41.20%, i.e., 11,198 children) could not use the mobile phone at school at all, not even during recess.

In relation to the use of mobile phones in school, we wanted to know the way children spent their break time, so we asked what pupils did during break time. Looking at the overall summary of most frequent break time activities, we found out that communication with peers dominated (85.24%). However, we do not know how the actual communication goes, i.e., what pupils actually talk about. However, a clear difference in the way of spending break time was visible between schools with and without mobile phone restrictions (Table 3 and Table 4).

Methodology comment: We divided the sample into two groups, to be compared to each other. Both groups included approximately the same number of respondents and when calculating percentage differences, we worked with relative frequencies. Respondents were allowed to give multiple answers at the same time, i.e., the child was, for instance, allowed to use a mobile phone when walking on the school premises. The following results show an overview of the most frequent break time activities.

In the research, we also differentiated between activities of children in the primary tier and first years of the secondary tier. The results are presented in the following charts (Table 5 and Table 6). The application of the chi-squared confirmed that the statistical differences between the responses obtained in the two types of centres are significant (*p* value < 0.001).

**Figure 1:**
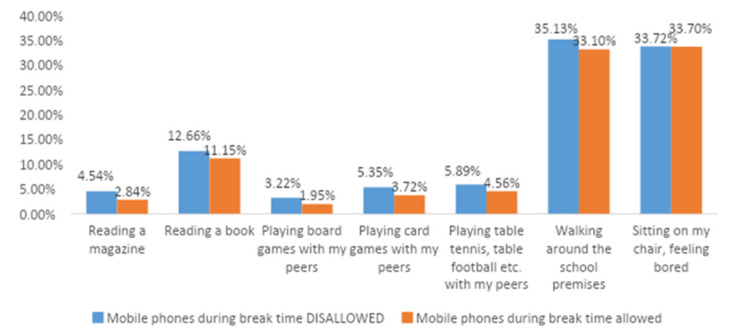
Activities carried out during break times depending on the availability of the mobile phone.

The difference is obvious—where mobile phones during break time were allowed, activities related to mobile phones clearly dominated (Figure 1). The top activity was playing games, preferred by over 40% children in schools where mobile phones were allowed during break time. The second place was taken by the use of social networks (almost 39%). In addition, a significant number of children passively watched their peer’s games or videos—22% more in schools where mobile phones were allowed, compared to schools that prohibited mobile phones.

Interestingly, approximately one third (33%) of children felt bored during break times (33%), regardless of mobile phones during break time being allowed or not. As for moving around the school premises, this was not influenced by mobile phones being allowed or restricted. In schools with a total mobile phone ban, approximately 6% more children walked around, compared to schools with mobile phones allowed.

Obviously, a mobile phone ban also affects the frequency of activities that are not directly related to mobile phones—reading, sport, non-virtual entertainment. In schools where mobile phones were banned during break time, the number of children reading magazines during break time was almost 60% higher than in schools where mobile phones were allowed. An increase was also obvious in reading books (+13.54% on schools with the ban), playing board games (+65%), playing card games (+43%) and sport activities (+29%). Banning mobile phones during break time has, therefore, a real impact on the development of such activities.

It has to be said that, although mobile phones during break time might be banned by a school, some children do not comply. For instance, 5% children played games on their mobile phones although gaming was not allowed, 5% also used social networks regardless of the ban in place, 4% chatted with other people although it was not allowed, etc. However, a significant decrease was present in all observed activities, in comparison with schools where mobile phones were allowed.

#### 3.1.2. Taking Photos/Videos by Peers without Their Consent

In relation to mobile phone restrictions in school, it is often pointed out that a mobile phone in school might be misused, for instance to picturing peers without their consent. Therefore, we wanted to know how many children had experienced, in school, that someone made photos/videos of them without consent—during break time, lesson, or a school event.

A total of 35.71% children (9706 children in our sample) confirmed that they had been photographed by a peer without consent and 22.5% children (6115 children in our sample) confirmed that they had been videoed by a peer without consent. It is clearly not a marginal issue.

## 4. Discussion

Nowadays, the use of mobile phones among young people in the Czech Republic has increased considerably. This has given rise to the concern of different education professionals to study the use of mobile phones inside and outside the school environment and the risks that exist as a result of this phenomenon.

Based on this idea, the present research aims to measure the behaviour of Czech children and adolescents with regard to the use of mobile phones. Thus, the results presented show a clear attitude of boredom in which young people do not find entertainment unless they have the technological tool at their disposal.

More than half of the children confirmed that they had permanent access to the Internet on their mobile phone, without having to rely on Wi-Fi (e.g., at school or in a library). They used their mobile phones most frequently to make/receive calls, write/send messages, watch YouTube videos, take photos, play games or listen to music. In this sense, a line is established that coincides with studies such as [9] or [10] with regard to how these activities are becoming increasingly important in the daily chores of children and young people, especially the use of social networks or the massive use of platforms such as YouTube [21,22].

Furthermore, there are also concerns about the possible risks of techno-addiction that this may cause in young Czechs [21,22]. Similarly, the large percentages expressed in the results of this study could have a possible impact on the lack of concentration and dispersion, which are directly related to school performance [26]. In this sense, the lines established coincide with other previous studies [28].

In turn, we focused on break time and explored the impact of the prohibition/permission of mobile phones on their activities. Most children were allowed to use mobile phones during recess at school and were not allowed to use them during lessons. Where mobile phones were allowed, the dominant activity was playing with them, using social media and being bored in the chair.

Interestingly, we found roughly the same number of bored children in schools where mobile phones were banned during recess. In schools where mobile phones were banned during recess, the dominant activity was walking around the school grounds, sitting on a chair, feeling bored and reading books, followed by sports activities and card games. The application of the chi-squared test confirmed the existence of significant differences between the two groups. This undoubtedly creates a debate around the current motivations surrounding young people’s entertainment. We find ourselves in a context in which the Internet and the use of mobile devices occupy a large percentage of young people’s overall entertainment throughout the day. Given this evidence, it is necessary for parents and educators to establish a debate about the motivations of young people and how to encourage healthy leisure and free time habits, as well as reducing the use of mobile devices [10].

In this respect, when comparing the two samples, in schools where mobile phones were banned during recess, the number of children reading magazines during recess was almost 60% higher than in schools where mobile phones were allowed. An increase in book reading, board games, card games and sports activities was also observed. Therefore, it could be indicated, in line with previous studies, that mobile phone use is useful and can have a functional use within the school, as stated in recent research [16]. However, it is necessary to regulate their use within the school, with the aim of promoting leisure, socialisation, sport or reading activities, which undoubtedly promote greater benefits for children and adolescents.

As a result of all these ideas, the aim is to share some ideas with educators and professionals in this field, providing a series of myths related to the use of mobile phones at school, as well as expressing the need to promote their proper use and regulate their use.

**A. Myths related to mobile phones in school**

There is a wide range of myths on mobile phones in the school environment, often shared by non-professionals, such as parents, who often do not understand how mobile phone restrictions work and to whom the bans actually apply. We will focus on the most widespread ones.

**Myth no. 1—A mobile phone ban in school means that phones are also banned during lessons.**

One of the most frequent arguments in discussing mobile phones in school is based on the premise that a phone is a tool that can be effectively used in education (as a method of delivery, source of information, etc.) and a mobile phone ban will deprive us of such benefits. This is obviously misunderstood—mobile phone restrictions in school apply to pupils’ activities outside the approval and instruction from their teacher! It does not apply to situations when a teacher asks pupils to take out their phones and use them actively for a learning task. At this point, a mobile phone becomes a learning tool and we should not worry about children using it in lessons. A mobile phone ban, in fact, does not restrict the teacher’s work, or the activities organised by the teacher. Smartphones can be therefore freely used for any meaningful activity organised by the teacher.

**Myth no. 2—The use of the phone as a safety measure for emergency situations.**

Myths presented mainly by parents include the belief that a phone can ensure their child’s safety in an emergency. Therefore, their child should always have a mobile phone ready to call for help while in school. First, it has to be said that safety and protection of children during school time is the responsibility of their school. It is the school that provides safety (and this must be ensured by the school management or governing body). If a pupil gets injured in school, it must be reported to a teacher/deputy/principal instead of phoning home. The incident is dealt with by the school, that must inform parents. Teachers could even go to prison if a child is harmed as a result of a teacher‘s negligence! For emergency situations, schools have crisis management policies in place, describing what to do with every specific problem (incident). Moreover, using a mobile phone in emergency without the teacher‘s knowledge is not desirable—it can raise panic among the parents contacted by the child, although nothing serious actually happened and it is only the child wanting to share their emotions with their parents. Similarly, a child can initiate an intervention by rescue services although the incident could have been sorted easily on the spot.

**Myth no. 3—A mobile phone in school is necessary for parents keeping their child permanently under control.**

This myth is closely related to the previous one. It has to be noted, again, that parents should NOT control their children during school time. This is the responsibility (and the related liability for any problems) of the school. If parents need to send an urgent message to their child, they can always contact the school office or the specific teacher. The contrasts in this discussion are interesting—parents want freedom for their children, but, at the same time, they want to control them strictly, even while the children are in someone else’s care.

**Myth no. 4—During break times, children should enjoy the freedom they do not have during lessons. By restricting mobile phones, we suppress their freedom.**

This argument is often used by those who do not realise that even break times between lessons have a purpose. These are not the child‘s “free time” but they constitute a part of the educational process. Break times are in place particularly for a short rest (both mind and body), preparation for the upcoming lesson, time for physiological needs and interpersonal communication necessary for the child‘s participation in a social group (simply “chit-chat and gossip”). A school is actually one of few places (except afterschool clubs and classes) where a child can socialise through direct contact, which cannot be replaced by online chatting and sharing. It helps to develop non-verbal communication skills (facial expression, gestures), conflict resolution and coping skills, mutual respect, but also physical characteristics (smell, physical ability), etc. These cannot be simulated through technology. Children get their free time after the last lesson or lunch every day. From that moment, they can use their mobile phones freely, within any limits set by parents. They can contact their parents while staying in an afterschool club (although regulation is desirable here as well).

**Myth no. 5—Banning mobile phones in school will result in the prohibition effect—phones will be used secretly.**

This has not been confirmed by schools that have been keeping strict limits for some time. A wisely managed school introduces a mobile phone ban along with new opportunities. To introduce a mobile phone ban on the premises, the school must have a sufficient range of relax and sport zones available (a place for ball games, table football, table tennis, board games, construction toys, chairs, books, chill-out zones, art and musical equipment, an open-air space for a walk, etc.).

The above-mentioned prohibition effect only accompanies the first stage of “going non-mobile“ (this stage, according to reports, lasts for about a month), similar to an addict’s withdrawal symptoms—pupils must suddenly entertain themselves, put some effort in establishing communication with others, make concessions, resolve conflicts and practice other social skills. Moreover, this can be difficult for someone who interacts mostly with electronics. The prohibition effect emerges almost exclusively in schools that do not have a sufficient offer of inspiring activities in place. Therefore, it is considered suboptimal to promote the prohibition of cell phones in Czech schools. In the same vein, it follows the same line of [33], in which the prohibition of mobile phones in the classroom is considered to be ineffective.

**B. Regulating the use of mobile phones in school**

Limiting the use of mobile phones during break times does not harm children at all—on the contrary, it has a significantly positive effect on them. It offers something that cannot be provided by family and technology—in particular, a chance to socialise directly (and the related set of skills to resolve conflicts, establish and maintain real friendship, manage their emotions, control their own behaviour, try out new opportunities, break rules and accept responsibility for it, succeed in arguments, etc.). However, it is always necessary for the school to provide space for meaningful free-time activities—chillout zones, sport zones, etc.

Regulating the use of mobile phones is desirable and meaningful, particularly in preschools and primary schools (up to 12 years of age). Later, the restrictions can be gradually eased out.

**C. Positive ways of using phones for learning**

Smartphones, tablets and other touchscreen mobile devices present a wide range of technologies that can be easily exploited in both school lessons and for homework. In addition, they are part of so-called BYOD (bring your own device) concept, where children actively use devices brought from home. Smartphones can be used for learning in several ways. The simplest method is presenting text, audio/video footages, animations, visualisations, school projects, digital learning programmes, etc., on the screens. Smartphones and tablets can be linked to interactive whiteboards and the learning content can be easily presented to the entire class.

Another option is to use touchscreen devices for electronic textbooks (ebooks). These allow, apart from simply presenting the learning topics, interaction with active elements (hypertext links, interactive tasks, etc.). In fact, many publishers now provide their classic paper textbooks along with an electronic version, extended by features, such as automatic evaluation of exercises and tasks or multimedia content that cannot be presented in a paper textbook. With modern electronic textbooks, teachers can simulate a range of processes and phenomena that are no longer demonstrated in schools today, such as autopsy of an animal, dangerous chemical reactions, insight into complex mechanisms, etc.

A smartphone or tablet can be also used for collaborative learning. There is a wide range of applications available, allowing users to share a whiteboard, create shared mental or terminology maps, or shared message boards, communicate with each other, vote in polls, test and internalise their knowledge through many test programmes, etc.

A smartphone or tablet can be naturally used as a gateway to the Internet and online services. Pupils can use it to search for and verify information, download content, watch videos, plan routes, communicate, vote, test their knowledge, etc. A smartphone can also be used as an augmented reality or mixed reality tool. Augmented reality allows inserting virtual elements (text, videos, 3D objects) into “real“ reality through a mobile touchscreen device.

Thanks to their mobility and battery life, mobile phones and tablets are great for research/discovery forms of learning. Watching and time-lapsing wildlife, determining fauna and flora species, tracking location (longitude/latitude, elevation), discovering the laws of nature, etc., can be all done with a smartphone. In this sense, this coincides with studies carried out in other countries that support the integration of these technological resources in the classroom, as well as their standardization within the school [17,18].

## 5. Conclusions

Czech youth find themselves in a situation where a large part of their leisure time at school is spent in contact with mobile phones. In cases where the use of these devices is prohibited on school grounds, it was reported that many of them were bored and did not know what to do.

From this work, we identified this pattern of behaviour among young Czechs, which is undoubtedly a concern for their personal and educational future. We find ourselves in a context in which technologies dominate most of young people’s time, leaving aside healthy lifestyle habits, such as sports. As a consequence, the rates of addiction to mobile phones and the Internet may increase, which are present and future threats in young people’s society. The aim of this work is to establish the facts around the question of the behaviour of Czech children and adolescents.

Furthermore, the primary education stage should serve as a preventive measure to promote responsible habits in children with regard to possible attitudes in adolescence [34] with respect to the use of mobile phones and the threats that exist on the Internet.

Therefore, we advocate the need to promote educational policies in educational centres, which, through a coordinated exercise with families, should promote the implementation of activities of various kinds to stimulate children to reduce the use of mobile phones. It is also considered that prohibiting their use in educational centres may not be the most effective option for achieving favourable results. Therefore, the integration of mobile phones in the classroom in a responsible way by educational centres could be a way to normalize their use in the classroom and prevent students from feeling the desire to use these devices in secret.

One of the limitations of this study is that it was not possible to find out more independent variables in the study sample, which would have provided some additional statistical input, as well as the relationships between the dependent and independent variables. Likewise, as a future line of research, it would be relevant to continue studying mobile phone use behaviour in other countries and to establish comparative studies between territories. Likewise, it would be interesting to focus on topics such as Internet or mobile phone addiction in order to provide more empirical evidence in this field of knowledge.

In conclusion, while technology has brought many benefits to society, its use in the context of youth needs to be regulated. Encouraging social and sporting practices by schools, in collaboration with families, will promote a healthier and less disease-prone environment. Moreover, encouraging responsible use of mobile phones will help this social group to take advantage of all the benefits offered by the Internet and its devices and to distance themselves from the threats that also reside in them.

## Figures and Tables

**Table 1:** Most frequent activities on a mobile phone.

Activity	Total Frequency (*n*)	Relative Frequency (%)
Making/receiving phone calls	19.701	72.49
Typing and sending messages on on-line services (Facebook Messenger, WhatsApp, etc.)	18.044	66.39
Watching videos on YouTube	17.778	65.42
Typing and sending SMS/MMS messages	14.735	54.22
Taking photos	14.039	51.66
Playing games	13.457	49.52
Listening to music or spoken audio (e.g., on Spotify, Apple Music, etc.)	12.801	47.10
Searching for information (e.g., on Google)	10.400	38.27
Watching favourite YouTubers	9091	33.45
Browsing social networks (passive, reading posts)	8811	32.42
Rating content on social networks (liking, rating by emoticons—such as Hearts on TikTok or Instagram).	8608	31.67
Sharing photos and videos on social networks	7005	25.78
Watching videos on TikTok	5319	19.57
Making videos	4702	17.30
Using a mobile phone for educational purposes (educational apps/videos/content)	4580	16.85
Reading texts on a mobile phone (e.g., text documents, books, PDF files, etc.)	3969	14.60
Managing a social network account (managing own wall, managing photo albums and video albums, creating campaigns)	3900	14.35
Watching videos on Twitch	3588	13.20
Streaming videos (e.g., through Twitch or Facebook)	1818	6.69

**Table 2:** Using mobile phones in school (from the children’s perspective).

Breaks	Lessons	Relative Frequency (%)
Allowed	Prohibited	53.30%
Prohibited	Prohibited	41.20%
Allowed	Allowed	2.48%
Prohibited	Allowed	1.09%
Not stated	Not stated	1.92%

**Table 3:** The most frequent break time activities—schools where mobile phones are allowed during break time.

Activities	Total Frequency (*n*)	Relative Frequency (%)
Playing games on my mobile phone	6216	41.00
Browsing social networks on my mobile phone	5898	38.90
Sitting on my chair, feeling bored	5110	33.70
Walking around the school premises	5018	33.10
Writing to someone on my mobile phone	4246	28.00
Listening to music on my mobile phone	4190	27.63
Watching my peers playing games/watching videos, etc., on their mobile phones	3364	22.19
Browsing websites on my mobile phone	2422	15.97
Watching YouTube videos on my mobile phone	2014	13.28
Reading a book	1690	11.15
Watching TikTok videos on my mobile phone	1270	8.38
Playing table tennis, table football, etc., with my peers	691	4.56
Making videos on my mobile phone	635	4.19
Playing card games with my peers	564	3.72
Reading a magazine	431	2.84
Playing board games with my peers	295	1.95

**Table 4:** The most frequent break time activities—schools where mobile phones are not allowed during break time.

Activities	Total Frequency (*n*)	Relative Frequency (%)
Walking around the school premises	4329	38.66
Sitting on my chair, feeling bored	3892	34.75
Reading a book	1667	14.89
Playing table tennis, table football etc. with my peers	857	7.65
Playing card games with my peers	835	7.46
Reading a magazine	748	6.68
Watching my peers playing games/watching videos, etc., on their mobile phones	627	5.60
Playing games on my mobile phone	611	5.46
Browsing social networks on my mobile phone	595	5.31
Playing board games with my peers	536	4.79
Writing to someone on my mobile phone	488	4.36
Listening to music on my mobile phone	451	4.03
Browsing websites on my mobile phone	246	2.20
Watching YouTube videos on my mobile phone	196	1.75
Watching TikTok videos on my mobile phone	162	1.45
Making videos on my mobile phone	130	1.16

**Table 5:** What do primary tier (7–11-year-old) children do during break times.

	Mobile Phones during Break Time ALLOWED		Mobile Phones during Break Time DISALLOWED			
Activity	Total Frequency (*n*)	Relative Frequency (%)	Total Frequency (*n*)	Relative Frequency (%)	χ2	*p* Value
Chatting with other pupils	1959	80.55	4146	87.88	167.94	***
Playing games on my mobile phone	930	38.24	92	1.95	146.37	***
Walking around the school premises	758	31.17	1557	33.00	141.94	***
Watching my peers playing games/watching videos, etc., on their mobile phones	686	28.21	164	3.48	124.56	***
Sitting on my chair, feeling bored	670	27.55	1330	28.19	120.97	***
Listening to music on my mobile phone	364	14.97	56	1.19	76.64	***
Browsing social networks on my mobile phone	337	13.86	36	0.76	70.84	***
Reading a book	300	12.34	879	18.63	67.76	***
Watching TikTok videos on my mobile phone	279	11.47	60	1.27	55.94	***
Watching YouTube videos on my mobile phone	217	8.92	46	0.97	41.64	***
Writing to someone on my mobile phone	214	8.80	30	0.64	46.64	***
Playing table tennis, table football, etc., with my peers	152	6.25	378	8.01	37.94	***
Reading a magazine	134	5.51	418	8.86	23.64	***
Browsing websites on my mobile phone	117	4.81	25	0.53	19.97	***
Playing card games with my peers	111	4.56	453	9.60	17.16	***
Playing board games with my peers	102	4.19	365	7.74	15.64	***
Making videos on my mobile phone	92	3.78	22	0.47	9.67	***
Watching YouTubers on my mobile phone	0	0.00	0	0.00	0	
Not stated	2432		4718			

Note: *** = *p* value < 0.001.

**Table 6:** What do first years of the secondary tier (12–15-year-old) children do during break times.

	Mobile Phones during Break Time ALLOWED		Mobile Phones during Break Time DISALLOWED			
Activity	Total Frequency (*n*)	Relative Frequency (%)	Total Frequency (*n*)	Relative Frequency (%)	χ2	*p* Value
Chatting with other pupils	7864	85.44	5785	88.15	345.27	***
Playing games on my mobile phone	3958	43.00	536	8.17	278.69	***
Browsing social networks on my mobile phone	3430	37.27	553	8.43	245.64	***
Sitting on my chair, feeling bored	3105	33.74	2546	38.79	237.95	***
Walking around the school premises	3247	35.28	2816	42.91	244.81	***
Writing to someone on my mobile phone	2316	25.16	446	6.80	246.97	***
Listening to music on my mobile phone	2573	27.96	408	6.22	256.94	***
Watching my peers playing games/watching videos, etc., on their mobile phones	2194	23.84	492	7.50	234.64	***
Browsing websites on my mobile phone	1272	13.82	224	3.41	202.64	***
Watching YouTube videos on my mobile phone	1188	12.91	155	2.36	197.82	***
Reading a book	960	10.43	805	12.27	185.64	***
Watching TikTok videos on my mobile phone	820	8.91	120	1.83	164.21	***
Playing table tennis, table football, etc., with my peers	442	4.80	495	7.54	101.64	***
Making videos on my mobile phone	418	4.54	106	1.62	97.64	***
Playing card games with my peers	339	3.68	393	5.99	81.64	***
Reading a magazine	230	2.50	358	5.45	59.64	***
Playing board games with my peers	135	1.47	187	2.85	27.46	***
Watching YouTubers on my mobile phone	0	0.00	0	0.00	0	
Not stated	9204		6563			

Note: *** = *p* value < 0.001.
